# Synthesis and Antimicrobial Activity of 5-Imidazolinone Derivatives

**DOI:** 10.4103/0250-474X.51953

**Published:** 2009

**Authors:** N. C. Desai, A. M. Bhavsar, B. B. Baldaniya

**Affiliations:** Medicinal Chemistry Division, University Department of Chemistry, Bhavnagar University, Bhavnagar-364 002, India

**Keywords:** 5-Imidazolinone, antibacterial activity, antifungal activity

## Abstract

Several 4-arylidene-2-phenyl-1-(2,4,5-trichlorophenyl)-1H-imidazol-5(4H)-ones (4a-q), N-(4-benzylidene-5-oxo-2-phenyl-4,5-dihydroimidazol-1-yl)-4-chlorobenzamides (5a-o) and N-(4-benzylidene-5-oxo-2-phenyl-4,5-dihydroimidazol-1-yl)-2,4-dichlorobenzamides (6a-m) were prepared. All newly synthesized compounds have been tested for their antibacterial activity against gram (+)ve and gram (−)ve bacteria and also on different strains of fungi. Introduction of OH, OCH_3_, NO_2_, Cl and Br groups to the heterocyclic frame work enhanced antibacterial and antifungal activities.

Imidazolinone ring system is of biological and chemical interest since long. The imidazolinones[1] are associated with a wide range of therapeutic activities[2–7] such as anticonvulsant, sedative and hypnotic, potent CNS depressant, antihistamine, antifilarial, bactericidal, fungicidal, antiinflammatory, MAO inhibitory, antiparkinsonian, antihypertensive and anthelmintic. Recently some new imidazolinone derivatives have been reported as antiinflammatory, herbicidal and hypertensive activities. Some workers have recognized 5-imidazolone as having anticancer activity[8]. The therapeutic importance of the compounds inspired us to synthesize some potential imidazolinones[9–13].

Desai *et al.*[14] have synthesized 4-benzylidene-2-phenyloxazole-5-one based on the methods descried in the literature which is a special type of Perkin condensation in which reaction between aldehyde and benzoylglycine proceeds first followed by ring closer. It is observed that aldehyde condenses under the influence of a base with the reactive methylene group in the azalactone which is formed by the dehydration of benzoylglycine, when the latter reacts with Ac_2_ O in presence of sodium acetate. In view of these observations, we have synthesized imidazol-5-ones (Scheme I, Table 1).

**Scheme 1:**
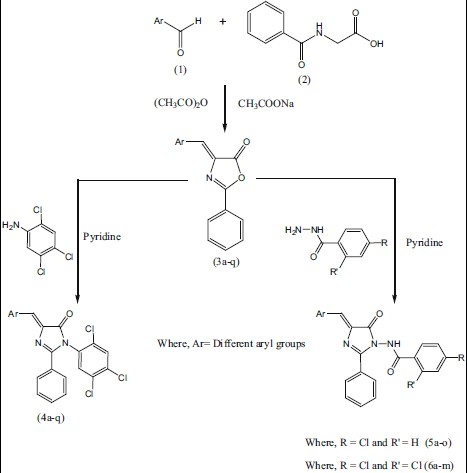
Synthetic pathway for synthesis of 5-imidazolone derivatives

**TABLE 1:** PHYSICAL CONSTANTS AND ELEMENTAL ANALYSIS OF 5-IMIDAZOLNES4a-q, 5a-o AND 6a-m

Sr No	Ar-	Molecular Formula	M.P.	Yield (%)	Elemental Analysis			
					
					% Carbon		% Nitrogen	
						
					Cal.	Found	Cal.	Found
4a	C_6_H_5_	C_22_H_13_Cl_3_N_2_O	173	65	61.78	61.69	6.55	6.41
4b	2-OH-C_6_H_4_	C_22_H_13_Cl_3_N_2_O_2_	170	60	59.55	59.47	6.31	6.25
4c	4-OCH_3_-C_6_H_4_	C_23_H_15_Cl_3_N_2_O_2_	160	55	60.35	60.28	6.12	6.03
4d	3-Cl-C_6_H_4_	C_22_H_12_Cl_4_N_2_O	185	54	57.17	57.06	6.06	6.01
4e	3-OCH_3_-C_6_H_4_	C_23_H_15_Cl_3_N_2_O_2_	190	50	60.35	60.21	6.12	6.03
4f	2-Cl-C_6_H_4_	C_22_H_12_Cl_4_N_2_O	195	53	57.17	57.06	6.06	5.98
4g	4-Cl-C_6_H_4_	C_22_H_12_Cl_4_N_2_O	210	54	57.17	57.05	6.06	6.01
4h	2-NO_2_-C_6_H_4_	C_22_H_12_Cl_3_N_3_O_3_	180	57	55.90	55.79	8.89	8.80
4i	3-NO_2_-C_6_H_4_	C_22_H_12_Cl_3_N_3_O_3_	230	55	55.90	55.74	8.89	8.78
4j	3-OCH_3_-4-OH-C_6_H_3_	C_23_H_15_Cl_3_N_2_O_3_	170	65	58.31	58.21	5.91	5.70
4k	5-Br-3OCH_3_-4-OH-C_6_H_2_	C_23_H_14_BrCl_3_N_2_O_3_	235	68	49.99	49.85	5.07	4.98
4l	4-OH-C_6_H_4_	C_22_H_13_Cl_3_N_2_O_2_	145	57	59.55	59.36	6.31	6.21
4m	5-Br-2OH-C_6_H_3_	C_22_H_12_BrCl_3_N_2_O_2_	175	50	50.56	50.45	5.36	5.22
4n	3-OC_6_H_5_-C_6_H_4_	C_28_H_17_Cl_3_N_2_O_2_	160	48	64.70	64.64	5.39	5.34
4o	2,4,5 (OCH_3_)3-C_6_H_2_	C_25_H_19_Cl_3_N_2_O_4_	198	45	57.99	57.90	5.41	5.33
4p	3,4,5 (OCH_3_)3-C_6_H_2_	C_25_H_19_Cl_3_N_2_O_4_	185	45	57.99	57.89	5.41	5.35
4q	3-OH-C_6_H_4_	C_22_H_13_Cl_3_N_2_O_2_	165	58	59.55	57.47	6.31	6.26
5a	C_6_H_5_	C_23_H_16_ClN_3_O_2_	233	60	68.74	68.62	10.46	10.35
5b	2-OH-C_6_H_4_	C_23_H_16_ClN_3_O_3_	235	65	66.11	66.05	10.06	9.97
5c	3-Cl-C_6_H_4_	C_23_H_15_Cl_2_N_3_O_2_	237	66	63.32	63.20	9.63	9.51
5d	3-OCH_3_-C_6_H_4_	C_24_H_18_ClN_3_O_3_	246	55	66.75	66.66	9.37	9.29
5e	2-Cl-C_6_H_4_	C_23_H_15_Cl_2_N_3_O_2_	212	62	63.32	63.19	9.63	9.51
5f	4-Cl-C_6_H_4_	C_23_H_15_Cl_2_N_3_O_2_	214	64	63.32	63.23	9.63	9.54
5g	2-NO_2_-C_6_H_4_	C_23_H_15_ClN_4_O_4_	248	50	61.82	61.73	12.54	12.45
5h	3-NO_2_-C_6_H_4_	C_23_H_15_ClN_4_O_4_	223	56	61.82	61.69	12.54	12.47
5i	3-OCH_3_-4-OH-C_6_H_3_	C_24_H_18_ClN_3_O_4_	226	45	64.36	64.25	9.38	9.25
5j	4-OH-C_6_H_4_	C_23_H_16_ClN_3_O_3_	231	47	66.11	66.01	10.06	9.98
5k	3-OC_6_H_5_-C_6_H_4_	C_29_H_20_ClN_3_O_3_	186	48	70.52	70.40	8.51	8.39
5l	2,4,5 (OCH_3_)3-C_6_H_2_	C_26_H_22_ClN_3_O_5_	245	46	63.48	63.40	8.54	8.47
5m	3,4,5 (OCH_3_)3-C_6_H_2_	C_26_H_22_ClN_3_O_5_	210	50	63.48	63.41	8.54	8.45
5n	3-OH-C_6_H_4_	C_23_H_16_ClN_3_O_3_	182	57	66.11	66.02	10.06	9.98
5o	4-N(C_2_H_5_)2-2-OH-C_6_H_3_	C_27_H_25_ClN_4_O_3_	176	43	66.21	66.21	11.46	11.40
6a	2-OH-C_6_H_4_	C_23_H_15_Cl_2_N_3_O_3_	175	60	61.08	61.01	9.29	9.93
6b	3-Cl-C_6_H_4_	C_23_H_14_Cl_3_N_3_O_2_	208	58	58.68	58.61	8.39	8.88
6c	3-OCH_3_-C_6_H_4_	C_24_H_17_Cl_2_N_3_O_3_	202	55	61.82	61.70	9.01	5.90
6d	2-Cl-C_6_H_4_	C_23_H_14_Cl_3_N_3_O_2_	205	56	58.68	58.58	8.93	8.85
6e	4-Cl-C_6_H_4_	C_23_H_14_Cl_3_N_3_O_2_	244	55	58.68	58.59	8.93	8.87
6f	2-NO_2_-C_6_H_4_	C_23_H_14_Cl_2_N_4_O_4_	216	58	57.40	57.31	11.64	11.55
6g	3-NO_2_-C_6_H_4_	C_23_H_14_Cl_2_N_4_O_4_	233	60	57.40	57.32	11.64	11.56
6h	3-OCH_3_-4-OH-C_6_H_3_	C_24_H_17_Cl_2_N_3_O_4_	237	48	59.77	59.65	8.74	8.65
6i	4-OH-C_6_H_4_	C_23_H_15_Cl_2_N_3_O_3_	236	45	61.08	61.01	9.29	9.22
6j	3-OC_6_H_5_-C_6_H_4_	C_29_H_19_Cl_2_N_3_O_3_	224	48	65.92	65.80	7.95	7.81
6k	2,4,5 (OCH_3_)3-C_6_H_2_	C_26_H_21_Cl_2_N_3_O_5_	238	43	59.33	59.27	7.98	7.90
6l	3,4,5 (OCH_3_)3-C_6_H_2_	C_26_H_21_Cl_2_N_3_O_5_	212	45	59.33	59.26	7.98	7.88
6m	3-OH-C_6_H_4_	C_23_H_14_Cl_2_N_3_O_3_	190	48	61.08	61.01	9.29	9.20

Various 4-arylidene-2-phenyl-1-(2,4,5-trichlorophenyl)-1*H*-imidazol-5(4*H*)-ones (4a-q) were prepared by the reaction of 2,4,5-trichlorobenzenamine with 4-arylidene-2-phenyloxazol-5(4*H*)-ones (3a-q). *N*-(4-benzylidene-5-oxo-2-phenyl-4,5-dihydroimidazol-1-yl)-4-chlorobenzamide (5a-o) were synthesized by the reaction of 4-chlorobenzohydrazide and 4-arylidene-2-phenyloxazol-5(4*H*)-ones (3a-q). *N*-(4-benzylidene-5-oxo-2-phenyl-4,5-dihydroimidazol-1-yl)-2,4-dichlorobenzamides (6a-m) were obtained by the reaction of 2,4-dichlorobenzohydrazide with 4-arylidene-2-phenyloxazol-5(4*H*)-ones (3a-q).

Melting points were taken in open capillaries using paraffin bath and are uncorrected. IR spectra were recorded on FTIR-Perkin-Elmer spectrometer (V_max_ cm^−1^); ^1^H NMR spectra were recorded on Bruker Avance 300 FT-NMR spectrometer using CDCl_3_ as a solvent and mass spectra carried out on JEOL SX 102/DA-600 mass spectrometer, respectively. All the compounds were analyzed for carbon, hydrogen and nitrogen and the results were within ±0.4% of theoretical values. Purity was checked by TLC using TLC aluminum sheets silica gel 60, supplied by E. Merck, Mumbai, India. The spots were located by keeping the plates in iodine vapor and 2,4,5-trichlorobenzenamine was supplied by S. D. Fine Chem Limited, Mumbai, India. 4-Chlorobenzohydrazide, 2,4-dichlorobenzo hydrazide and 4-arylidene-2-phenyloxazol-5(4*H*)-one (3a-q), were prepared as given in literature method[15–20].

4-Arylidene-2-phenyl-1-(2,4,5-trichlorophenyl)-1*H*-imidazol-5(4*H*)-one (4) were synthesized as follows; A mixture of 2,4,5-trichloroaniline (0.01 mol) and 4-(arylidene)-2-phenyloxazol-5(4*H*)-ones (0.01 mol) was placed in a round bottom flask and 10 ml of pyridine were added to it. The reaction mixture was refluxed on a sand bath for 6 h. (scheme I) and the mixture was poured into ice-cold water and then a required amount of conc. hydrochloric acid was added to neutralize the reaction mixture. The progress of the reaction and the purity of compounds were routinely checked on TLC. The solid obtained was left overnight, filtered and washed with water. The product was dried and recrystallized from ethanol (99%). m.p.195° Yield 53% anal. found: C, 57.06; N, 5.98; calc for C_22_ H_12_ Cl_4_ N_2_ O: C, 57.17; N, 6.06%.

Compound 4f: IR (KBr): 3062 cm^−1^ (-C-H str., aromatic), 1643 cm^−1^ (>C=O str., cyclic ring), 1359 cm^−1^ (>C=N str., imidazol ring), 1284 cm^−1^ (-C-N tertiary amine), 1074 cm^−1^ (-C-Cl str., aromatic), 744 cm^−1^ (>C=CH medium), 704, 688, 613 cm^−1^ (trisubstituted aromatic). ^1^H NMR (CDCl_3_): δ7.2 (s, 1H, -CH), 7.26-8.54 (m, 11H, Ar-H, C=C-Ar) ppm. MS: m/z 461 with 62% relative intensity (base peak) & 462 with 47% relative intensity (M^+^). Other compounds of the series were prepared by using a similar method and their physical data are recorded in Table 1.

*N*-(4-benzylidene-5-oxo-2-phenyl-4,5-dihydroimidazol-1-yl)-4-chlorobenzamides (5)/ *N*-(4-benzylidene-5-oxo-2-phenyl-4,5-dihydroimidazol-1-yl)-2,4-dichlorobenzamides (6) were prepared using the following procedure; A mixture of 4-chlorobenzohydrazide/ 2,4-dichlorobenzohydrazide (0.01 mol) and 4-(arylidene)-2-phenyloxazol-5(4*H*)-ones (0.01 mol) was placed in a round bottom flask and 10 ml of pyridine was added to this mixture. The reaction mixture was refluxed on a sand bath for 6 h (Scheme I). The mixture was poured into ice-cold water and then required amount of con. hydrochloric acid was added to neutralize the reaction mixture. The solid obtained was left overnight, filtered and washed with water. The product was dried and recrystallized from ethanol (99%).

Compound 5f: IR (KBr): 3249 cm^−1^ (medium –CONH-), 3033 cm^−1^ (-C-H str., aromatic), 1656 cm^−1^ (>C=O str., cyclic ring), 1625 cm^−1^ (>C=N str., imidazol ring), 1490 cm^−1^ (>NH weak), 1299 cm^−1^ (-C-N tertiary amine), 1095 cm^−1^ (-C-Cl str., aromatic), 754 cm^−1^ (>C=CH medium), 707 cm^−1^ (monosubstituted aromatic). ^1^H NMR (CDCl_3_): δ7.28 (s, 1H, -CH), 7.26-8.54 (m, 13H, Ar-H, -C=C-Ar), 10.02 (s, 1H, -NH-CO-) ppm. MS: m/z 436 with 45% relative intensity (base peak) & 437 with 32% relative intensity (M^+^).

Compound 6e: IR (KBr): 3213 cm^−1^ (medium, –CONH-), 2993 cm^−1^ (-C-H str., aromatic), 1662 cm^−1^ (>C=O str., cyclic ring), 1635 cm^−1^ (>C=N str., imidazol ring), 1473 cm^−1^ (>NH weak), 1305 cm^−1^ (-C-N tertiary amine), 1109 cm^−1^ (-C-Cl str., aromatic), 925 cm^−1^ (>C=CH medium), 825, 713 cm^−1^ (disubstituted aromatic), 707 cm^−1^ (monosubstituted aromatic). ^1^H NMR (CDCl_3_): δ7.2 (s, 1H,-CH), 7.32-8.05 (m, 12H, Ar-H, C=C-Ar), 10.02 (s,1H, -NH-CO-) ppm. MS: m/z 471 with 79% relative intensity (base peak) and 472 with 51% relative intensity (M^+^). Other compounds of the series were prepared by using a similar method and their physical data are recorded in Table 1.

Antibacterial activity was carried out by broth dilution method[21]. The strains used for the activity were procured from Institute of Microbial Technology, Chandigarh. The compounds 4a-q, 5a-o and 6a-m were screened for their antibacterial activity against *Escherichia coli, Staphylococcus aureus, Pseudomonas aeruginosa* and *Staphylococcous pyogenes* at concentrations of 1000, 500, 200, 100, 50, 25, 12.5 µg/ml respectively (Table 2). Same compounds were tested for antifungal activity against *C. albicans, A. niger* and *A. clavatus* at concentrations of 1000, 500, 200, and 100 µg/ml respectively (Table 2). The results are recorded in the form of primary and secondary screening.

**TABLE 2:** ANTIBACTERIAL AND ANTIFUNGAL ACTIVITIES OF THE SYNTHESIZED COMPOUNDS*

Sr. No.	Minimal bactericidal concentration (MBC) in μg/ml				Minimal fungicidal concentration (MFC) in μg/ml		
	
	*E. coli* MTCC-443	*P. aeruginosa* MTCC-1688	*S. aureus* MTCC-96	*S. pyogenus* MTCC-442	*C. albicans* MTCC-227	*A. niger* MTCC-282	*A. clavatus* MTCC-1323
4a	25	-	-	-	-	-	-
4b	25	50	-	-	-	-	-
4f	100	-	-	-	100	100	100
4i	25	-	-	-	-	-	-
4j	25	-	-	-	-	-	-
4k	50	100	50	-	-	-	-
4q	50	100	-	-	-	-	-
5b	-	-	-	-	100	100	100
5e	50	50	-	-	-	-	-
6f	100	-	-	-	100	100	100
6j	-	-	-	-	-	-	100
6l	-	-	-	-	100	-	100
6m	-	-	-	-	100	-	-

Gentamycin is used as standard for antibacterial activity which showed (0.05, 0.25, 0.5 and 1 μg/ml) MBC against *E. coli*, *S. aureus*, *S. pyogenus* and *P. aeruginosa* respectively. K nystatin was used as the standard for antifungal activity which showed 100 μg/ml MFC against fungi, used for the antifungal activity.

* All the compounds were tested for the antibacterial and antifungal activities but data of active compounds have been reported as present protocol

The synthesized compounds found to be active in the primary screening were further tested in a second set of dilution against all microorganisms. The compounds found active in primary screening were similarly diluted to obtain 100, 50, 25 μg/ml concentrations. Ten microlitres suspensions from each well were further inoculated on appropriate media and growth was noted after 24 and 48 h. The lowest concentration, which showed no growth after spot subculture was considered as MBC/MFC for each drug. The highest dilution showing at least 99% inhibition was taken as MBC/MFC. The result of this test is affected by the size of the inoculums. The test mixture should contain 10^8^ organisms/ml. For antibacterial activity, in present protocol 50 µg/ml is considered as active as compared to the standard drug gentamycin. For antifungal activity, 100 µg/ml is considered as active as compared to standard nystatin. Compounds 4a, 4b, 4i, 4j, 4k, 4q and 5e are active on *E. coli* where as 4b and 5e are active on *P. aeruginosa*. Compound 4k is active on *S. aureus* and 6m is also active on *S. pyogens.* Compounds 4f, 5b, 6f, 6l, and 6m are active on fungi strains. On the basis of biological activity results, it may be concluded that the introduction of OH, OCH_3_, NO_2_, Cl and Br groups to the heterocyclic frame work enhanced antibacterial and antifungal activities.
